# Lnc-TCL6 is a potential biomarker for early diagnosis and grade in liver-cirrhosis patients

**DOI:** 10.1093/gastro/goz050

**Published:** 2019-10-11

**Authors:** Lei-Jia Li, Xiao-Ying Wu, Si-Wei Tan, Zi-Jun Xie, Xue-Mei Pan, Shun-Wen Pan, Wu-Ri-Na Bai, Hai-Jiao Li, Hui-Ling Liu, Jie Jiang, Bin Wu

**Affiliations:** 1 Department of Gastroenterology, The Third Affiliated Hospital of Sun Yat-sen University, Guangzhou, Guangdong, P. R. China; 2 Guangdong Provincial Key Laboratory of Liver Disease Research, Guangzhou, Guangdong, P. R. China; 3 Department of Laboratory Medicine, The Third Affiliated Hospital of Sun Yat-sen University, Guangzhou, Guangdong, P. R. China

**Keywords:** long non-coding RNAs, Lnc-TCL6, biomarker, liver cirrhosis, Child–Pugh classification

## Abstract

**Background:**

Long non-coding RNAs (lncRNAs) have been applied as biomarkers in many diseases. However, scarce biomarkers are available in single lncRNA differential expression associated with different clinical stages of liver cirrhosis (LC). The aim of the study is to identify some lncRNAs that can serve as non-invasive sensitive biomarkers for early diagnosis and grade of LC.

**Methods:**

Blood lncRNA expression was evaluated in three independent cohorts with 305 participants including healthy controls, hepatitis B virus (HBV) carriers, and patients with chronic hepatitis B (CHB) or LC. First, candidate lncRNAs were screened by CapitalBiotech microarray to diagnose cirrhosis. Quantitative reverse-transcriptase polymerase chain reaction was then used to investigate the expression of selected lncRNAs in the whole group of cirrhosis and different Child–Pugh classes. Ultimately, the diagnostic accuracy of the promising biomarker was examined and validated via Mann–Whitney test and receiver-operating characteristics analysis.

**Results:**

Lnc-TCL6 was identified as a sensitive biomarker for early diagnosis of LC (Child–Pugh A) compared with healthy controls (area under the ROC curve [AUC] = 0.636), HBV carriers (AUC = 0.671), and CHB patients (AUC = 0.672). Furthermore, lnc-TCL6 showed a favourable capacity in discriminating among different Child–Pugh classes (AUC: 0.711–0.837). Compared with healthy controls, HBV carriers, and CHB patients, the expression of lnc-TCL6 was obviously up-regulated in Child–Pugh A patients and, conversely, significantly down-regulated in Child–Pugh C patients.

**Conclusions:**

Lnc-TCL6 is a novel potential biomarker for early diagnosis of LC and is a possible predictor of disease progression.

## Introduction

Advanced liver cirrhosis (LC) is currently a common cause of death [1]. LC is one of the top 20 causes of disability and loss of life, accounting for 1.6% of the worldwide burden [2]. The global prevalence of chronic hepatitis B (CHB) is 3.5% and the prevalence is more than 8% in Asia and Africa [3]. Due to the lack of standardized definition of LC and unified evaluation methods (non-invasive markers, laboratory examination, imaging examination, and biopsy), clinical and drug studies on LC are limited, and many patients with viral hepatitis and compensatory LC have missed the optimal time for diagnosis and treatment, thus developing decompensated cirrhosis and even liver failure.

The number of protein-coding genes in humans is <2% of the total genome sequence and the vast majority of transcripts consist of long non-coding RNAs (lncRNAs). Although lncRNAs are less conserved and expressed at a lower level than protein-coding genes, they are usually regulated by transcription factors and expressed in a cellular- or tissue-specific manner [4–6]. LncRNAs are involved in a variety of physiological-regulation and pathological processes and have become an effective diagnostic tool in many diseases [7–10]. Accumulating studies have attempted to reveal expression characteristics and various pro-fibrogenic pathways of lncRNA involved in liver fibrosis [11–14]. However, thus far, these studies have lacked accurate expression of lncRNA in LC tissues or peripheral blood. To fill this gap, our study aims to verify a potential lncRNA biomarker for early onset and predict the progress of hepatitis B virus (HBV)-related LC. In this study, the lncRNA-expression profile from a large cohort of patients with HBV-related LC, patients with HBV infection, and healthy people will be comparably evaluated. The expression characteristics of lncRNAs in different LC subgroups were statistically investigated. Finally, a potential non-invasive lncRNA biomarker for early onset and grade of HBV-related LC was identified.

## Materials and methods

### Study population

In this study, we collected 305 whole-blood samples eligible for inclusion according to the qualification criteria from the Third Affiliated Hospital of Sun Yat-sen University from March 2017 to September 2018, among which 136 samples were from patients with HBV-related LC, 84 from HBV infection, and 85 from healthy people.

The healthy controls were negative for health-examination findings, including routine blood analyses, hepatitis markers, chest X-ray, abdominal ultrasound, routine stool and urine analyses, human immunodeficiency virus, Epstein-Barr virus, hepatitis C virus, and syphilis antibodies, and had no other chronic liver diseases such as autoimmune hepatitis, alcoholic liver disease, non-alcoholic fatty liver disease, or malignancy.

Inactive HBsAg carriers (HBV carriers) and CHB patients were included in the HBV-infection group; the former was defined according to the absence of significant necroinflammation with positive HBsAg over 6 months, serum HBV DNA <2,000 IU/mL, and persistently normal alanine aminotransferase (ALT) and/or aspartate aminotransferase (AST) levels. The diagnostic criteria for CHB were chronic liver inflammation with the presence of HBsAg for at least 6 months, serum HBV DNA levels generally >20,000 IU/mL in HBeAg-positive CHB (2,000–20,000 IU/mL in HBeAg-negative CHB), and intermittent or persistent elevation of ALT and/or AST levels [15].

Liver biopsy/hepatic ultrasound with computerized tomography (CT) or magnetic resonance imaging (MRI) was used to confirm the diagnosis of HBV-related LC. Child–Pugh classification was conducted using a scoring system summing the scores of serum total bilirubin (TBiL), serum albumin (ALB), prothrombin time (PT), ascites, and hepatic encephalopathy to grade LC as class A (CP-A, 5–6 points), B (CP-B, 7–9 points), or C (CP-C, 10 points or above) [16].

### Study design

**Figure 1.**
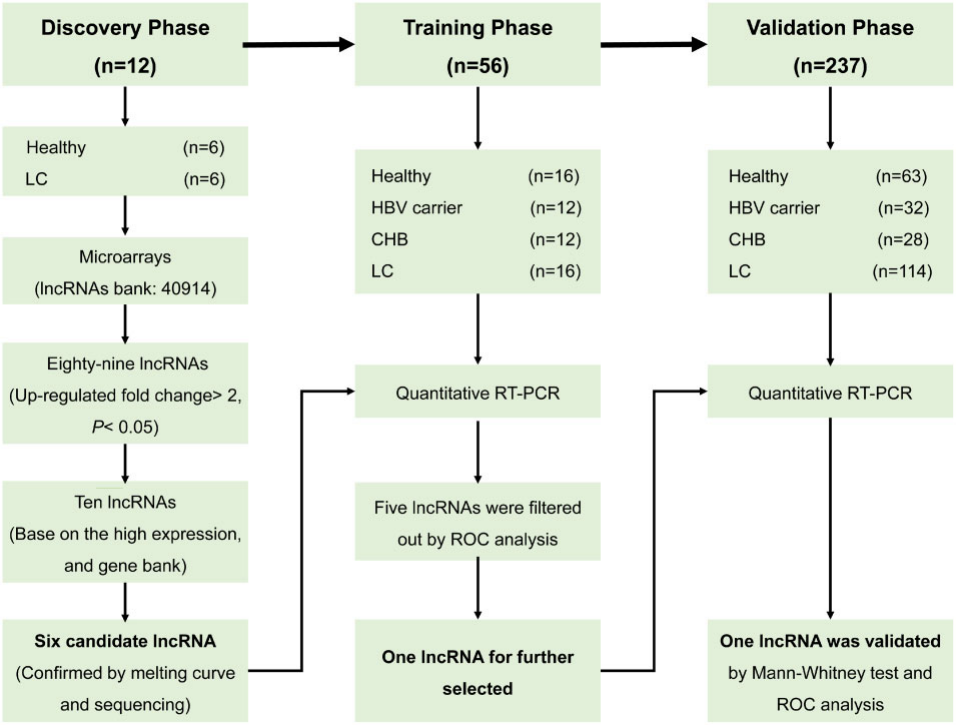
Study design. This study had three phases: the discovery phase, the training phase, and the validation phase. The main aim was to explore the candidate lncRNAs for HBV-related liver cirrhosis (LC) and validate the predictive value of lncRNA biomarkers in the early diagnosis and prognosis of LC.

The above samples were divided into three stages in chronological order (Figure 1). In the discovery phase, 12 whole-blood samples from volunteers were randomly selected, including six patients with HBV-related LC and six healthy controls (evenly split by sex). Each of these samples with 40,914 lncRNAs were analysed by lncRNA and mRNA array to select the differently expressed lncRNAs between the two groups with threshold values of ≥2- and ≤–2-fold change and a *t*-test *P*-value of 0.05. We chose the potential candidate biomarkers according to the following criteria: high relative expression values, not previously reported in other hepatic or sclerosing diseases, present in a reliable database and lncRNAs with no annotations excluded. Then, 10 genes were then selected for further detection by quantitative reverse-transcriptase polymerase chain reaction (qRT-PCR). Finally, candidate genes were screened by validation sequencing and agarose electrophoresis.

In the training phase, the lncRNAs initially screened by microarray were further tested in the 32 training participants (16 LC patients and 16 healthy controls) and the logistic-regression model was used to further identify the target lncRNAs with up-regulated expression in cirrhosis compared with the healthy controls. To explore the differential expression of candidate genes in the course of normal liver to HBV infection to cirrhosis, qRT-PCR was performed to discover the expression of the candidate lncRNAs in six pairwise comparisons: healthy controls vs HBV carriers, HBV carriers vs CHB group, healthy controls vs CHB group, healthy controls vs LC group, HBV carriers vs LC group, and CHB group vs LC group.

In the validation phase, the target genes selected from the training phase were applied to an independent cohort of 237 samples with the same comparison model as the training set. The differential expression of lncRNAs in CP-A, CP-B, and CP-C cirrhosis was further explored in respective analyses.

This study was conducted in accordance with the Declaration of Helsinki and the protocol was approved by the Ethics Committee of the Third Affiliated Hospital of Sun Yat-sen University (Ethical Number: 2017–2-248). Written consent for the information to be stored in the hospital database and used for research was obtained from the study participants.

### Procedures

The lncRNA and mRNA Human Gene array data were analysed for data summarization, normalization, and quality control using the GeneSpring software V13.0 (Agilent) in the discovery phase. The microarray data were Log2 transformed and median centred by genes using the Adjust Data function of CLUSTER 3.0 software then further analysed with hierarchical clustering with average linkage [17].

Whole-blood samples from 85 healthy controls, 44 HBV carriers, 40 CHB patients, and 136 LC patients were collected and stored at –80°C in 1.5-mL RNase-free microcentrifuge tubes for later use within 7 days. Total RNA was extracted and cDNAs were synthesized as previously described [18]. QRT-PCR of lncRNAs was performed using the ChamQ^TM^ SYBR qRT-PCR Master mix (Vazyme biotech, Nanjing, China) and quantified using an ABI 7500 Real-Time PCR System (Life Technologies, USA) with the validated specific primer sets. Primer sequences are listed in Supplementary Table 1. The reaction mixtures were pre-degenerated at 95°C for 30 s, followed by 40 cycles of 95°C for 10 s and 60°C for 30 s. The expression levels of candidate genes were normalized to the respective β-actin expression and each sample was analysed in triplicate. The specificity of each PCR was confirmed by melting curve analyses and Ct values >35 were excluded from the analyses. Data analyses were performed using the 2^−ΔΔCt^ method.

### Statistical analysis

For microarray analysis, the Mann–Whitney unpaired test was used for the two pairwise comparisons (LC patients vs healthy controls). Chi-square tests were used to test the characteristic differences between the training phase and validation phase. Continuous variables consistent with normal distribution were described as means ± the standard deviation (SD) and compared using ANOVA (Analysis of Variance). Continuous variables do not comply with the normal distribution using median [interquartile range] and compared using the Mann–Whitney *U* or Kruskal–Wallis *H* test with confounding factors such as age and sex used as covariates in the models.

Defining the occurrence of different Child–Pugh classes of cirrhosis as the state variable, we employed a receiver-operating characteristic (ROC) curve to analyse the predictive value of each significantly different factor between groups. The area under the ROC curve (AUC) was used as an accuracy index to evaluate the diagnostic performance of the candidate lncRNAs [19]. AUC was adjusted for covariate effects by regression modelling of the relationship between the marker and the covariates such as age and sex. MedCalc (version 10.4.7.0; MedCalc, Mariakerke, Belgium) software was used to perform the ROC and regression analysis. All other analyses were conducted with SPSS 21.0 (SPSS, Inc., Chicago, IL, USA). Two-tailed *P*-values <0.05 were considered statistically significant.

## Results

### Discovery phase: microarray profiling of lncRNAs associated with HBV-related LC

**Figure 2.**
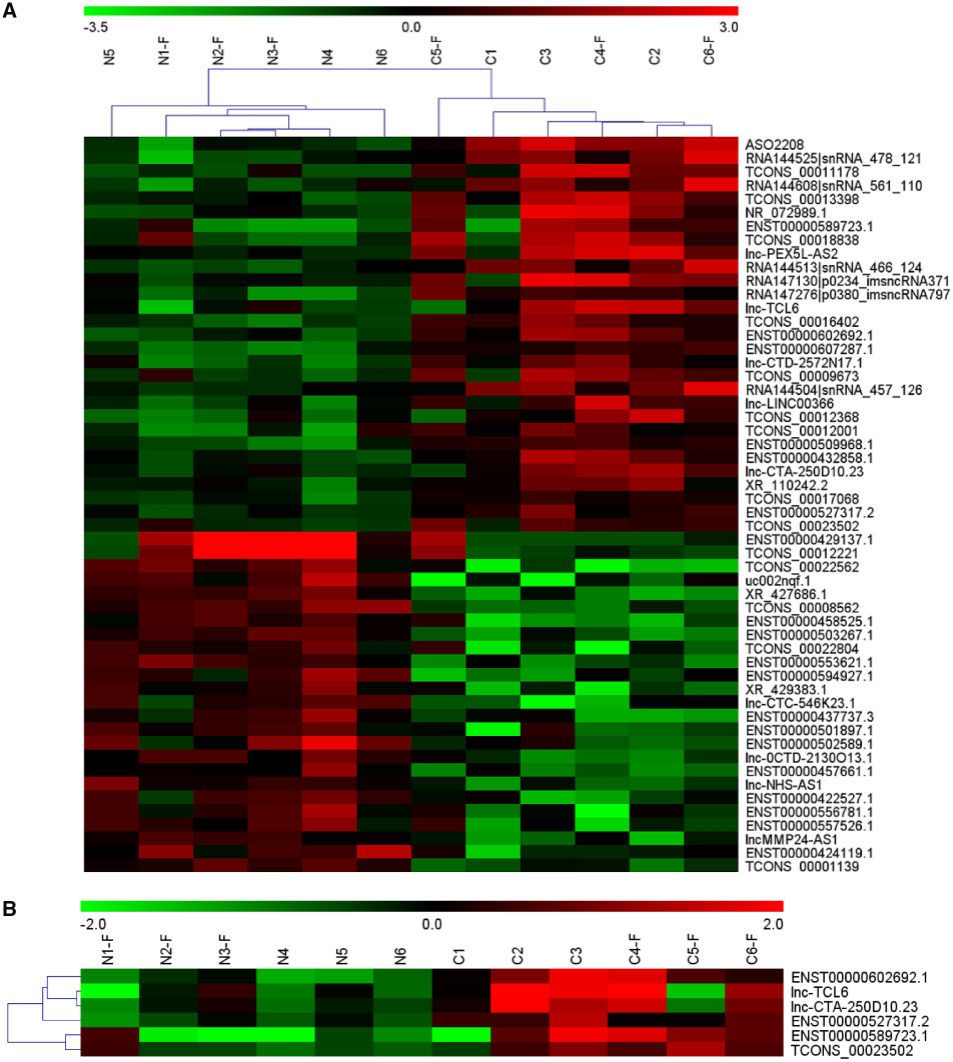
Microarray analysis of candidate lncRNA biomarkers in two group comparisons. (A) Numerous differentially expressed lncRNAs in whole blood from HBV-related liver-cirrhosis patients compared with the healthy group. (B) According to the data summarization and analysis of the microarray, ENST00000602692.1, ENST00000589723.1, lnc-CTA-250D10.23, ENST00000527317.2, TCONS_00023502, and lnc-TCL6 had significantly higher expression levels in the cirrhosis group than in the healthy group.

A microarray containing probes for 40,914 human lncRNAs was initially used to screen the significant differential expression levels of lncRNAs between the HBV-related LC and healthy controls; 149 lncRNAs were significantly up-regulated (fold change ≥2) and 126 lncRNAs were significantly down-regulated (fold change ≤–2) in the LC group. The raw microarray data and detailed instructions on the microarray have been uploaded to the Gene Expression Omnibus (Access number GSE123932). Figure 2A illustrates some of the numerous differentially expressed lncRNAs in the whole-blood samples from LC patients compared with the healthy group. According to the analysis of the microarray and data summarization, ENST00000602692.1, ENST00000589723.1, lnc-CTA-250D10.23, ENST00000527317.2, TCONS_00023502, and lnc-TCL6 were identified as candidate biomarkers with significantly higher expression levels in the LC group than in the healthy group (fold changes = 3.35, 3.67, 2.43, 2.07, 2.05, and 3.36, respectively, *P *<* *0.05; Figure 2B).

### Training phase: six potential biomarkers were selected for HBV-related LC

**Figure 3.**
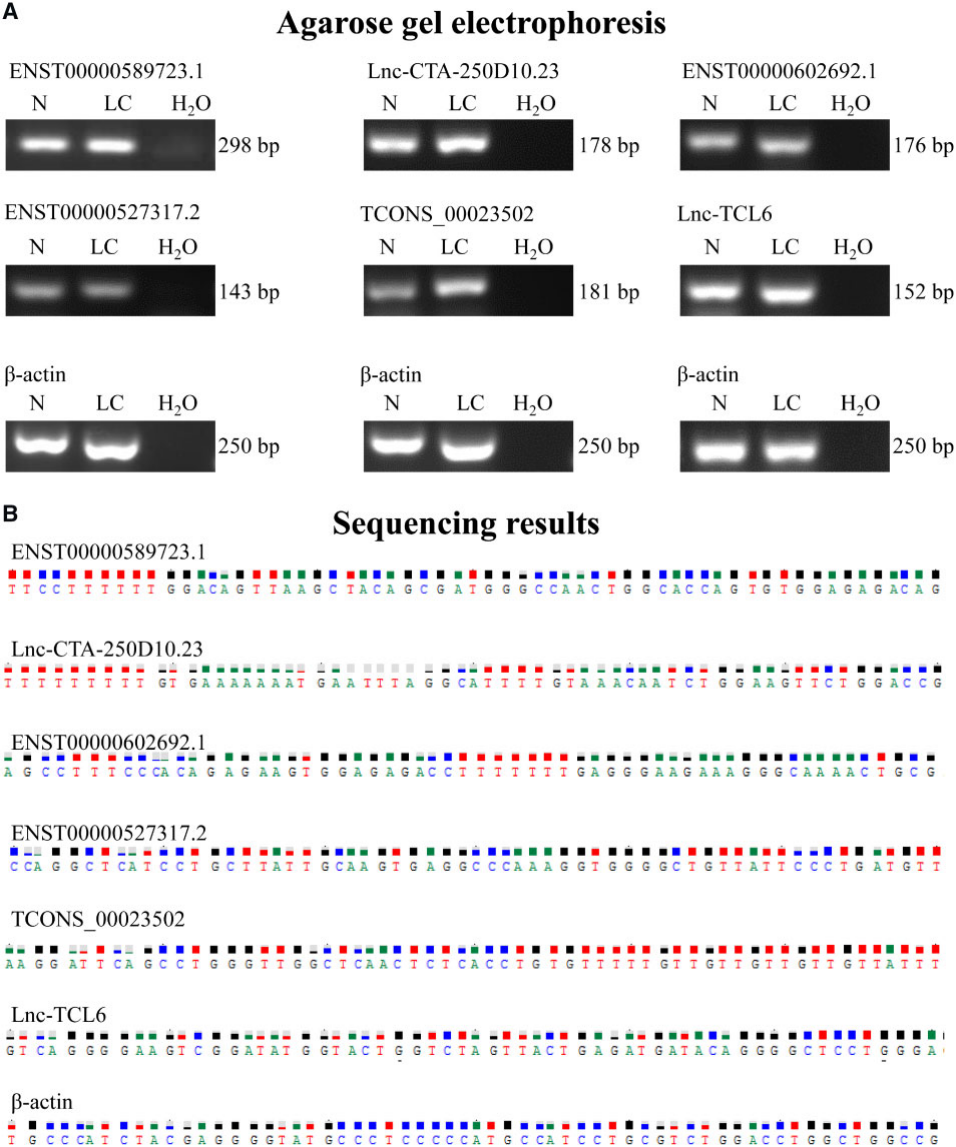
Agarose gel electrophoresis and sequencing results of six candidate lncRNAs. (A) PCR products of the candidate lncRNAs could be readily resolved by agarose gel electrophoresis. (B) Sequencing results showed that the expression of candidate lncRNAs in the whole blood of patients with HBV-related liver cirrhosis could be specifically detected by qRT-PCR in the training phase.

To confirm the results in the discovery set, the expression profiles of these six lncRNAs were evaluated via qRT-PCR on 32 additional blood samples (16 from healthy controls and 16 from LC patients). The results of sequencing and agarose gel electrophoresis are shown in Figure 3. The diagnostic accuracy of these six candidate lncRNA predictions in LC was assessed using ROC curve analysis. The corresponding AUCs were 0.141, 0.438, 0.525, 0.438, 0.438, and 0.762, respectively (Supplementary Table 2). According to the results, lnc-TCL6 showed good diagnostic value for the LC group compared with healthy controls.

**Figure 4.**
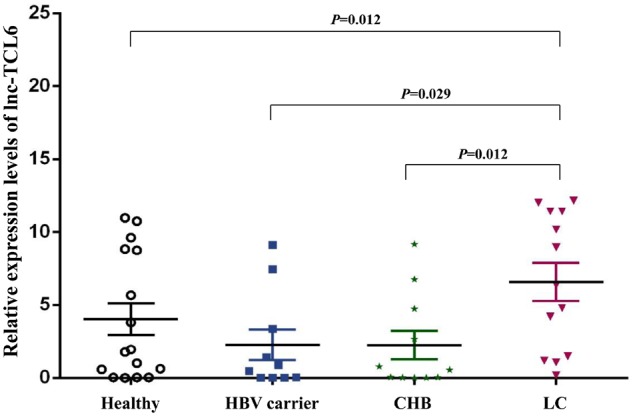
Relative expression levels of lnc-TCL6 in the training set. Compared with healthy controls, HBV carriers, and chronic hepatitis B (CHB) patients, the expression of lnc-TCL6 was significantly up-regulated in whole blood from HBV-related liver cirrhosis (LC) patients. Data are presented as mean with standard error.

In addition, we collected 24 whole-blood samples from HBV-related patients (the CHB group and the HBV carrier group each accounted for half; Table 1) and further tested the expression levels of lnc-TCL6 in six pairwise comparisons (healthy controls vs HBV carrier group, HBV carrier group vs CHB group, healthy controls vs CHB group, healthy controls vs LC group, HBV carrier group vs LC group, and CHB group vs LC group). The corresponding AUCs were 0.542, 0.556, 0.602, 0.762, 0.761, and 0.781, respectively (Table 2). The relative expression level of lnc-TCL6 measured by qRT-PCR showed a 1.63-fold increase in patients with LC compared with healthy controls, a 2.92-fold increase compared with the CHB group, and a 2.89-fold increase compared with HBV carriers (Figure 4). These data suggested that lnc-TCL6 could be used as an effective biomarker for predicting LC from healthy and HBV-infection groups but a poor diagnostic marker for HBV-infection groups compared with healthy individuals. Hence, lnc-TCL6 was chosen as a potential blood predictor for LC and measured further in larger sample sizes.

**Table 1.** Characteristics of research participants in the training phase

Variable	Healthy (*n* = 16)	HBV carrier (*n* = 12)	Chronic hepatitis B (*n* = 12)	Liver cirrhosis (*n* = 16)
Age, years	41.9 ± 14.9	43.4 ± 12.9	40.2 ± 12.5	51.6 ± 9.1
Female gender	7 (43.7)	6 (50.0)	3 (25.0)	3 (18.7)
White blood cell, × 10^9^/L	7.1 ± 1.5	5.7 ± 1.1	6.1 ± 1.3	4.9 ± 1.9
Alanine aminotransferase, U/L	16.0 [11.5–24.0]	31.5 [23.8–46.0]	208.0 [74.0–560.8]	28.0 [21.0–37.8]
Serum albumin, g/L	48.1 ± 2.0	40.7 ± 5.2	37.6 ± 5.8	35.5 ± 5.5
Total bilirubin, µmol/L	9.8 [7.5–11.0)]	11.1 [7.2–13.8]	71.9 [65.3–86.0]	25.1 [13.3–53.3]

Values presented as mean ± standard deviation, median [interquartile range], or *n* (%).

**Table 2.** The diagnostic value of lnc-TCL6 in the training set

Comparison	AUC	95% CI	P-value	Sensitivity	Specificity
Healthy vs HBV carrier	0.542	0.344–0.730	0.726	0.417	0.750
HBV carrier vs CHB	0.556	0.341–0.756	0.650	0.583	0.583
Healthy vs CHB	0.602	0.400–0.780	0.377	0.500	0.750
Healthy vs LC	0.762	0.579–0.894	0.002	0.438	1.000
HBV carrier vs LC	0.761	0.546–0.889	0.010	0.800	0.667
CHB vs LC	0.781	0.585–0.914	0.004	0.938	0.583

AUC, area under the curve; CI, confidence interval; CHB, chronic hepatitis B; LC, liver cirrhosis.

### Validation phase: independent validation of lnc-TCL6 in HBV-related LC

**Figure 5.**
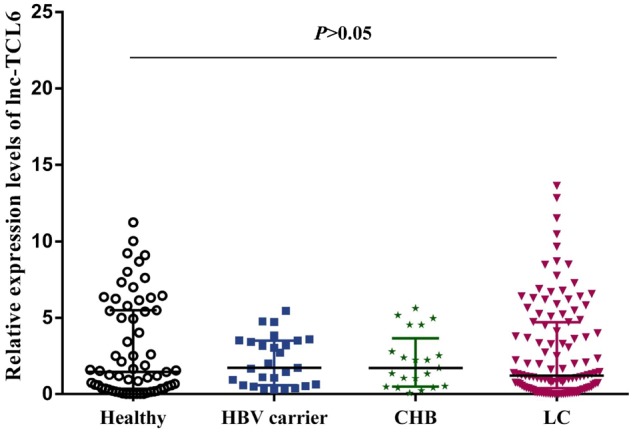
The expression profile of lnc-TCL6 in the validation phase. The relative expression level of lnc-TCL6 was similar among the healthy controls, HBV carriers, chronic hepatitis B (CHB) patients, and liver-cirrhosis (LC) patients. Data are presented as median with interquartile range.

The candidate lnc-TCL6 estimated from the training data set was further independently validated by qRT-PCR with blood samples obtained from 237 participants including 114 patients with HBV-related LC, 28 CHB patients, 32 HBV carriers, and 63 healthy controls. There were significant differences in the distribution of age, sex, WBC, ALB, and TBiL among the four groups (Table 3). In the LC group, the expression level of lnc-TCL6 was similar to that in the healthy group, HBC carriers, and CHB patients (all *P *>* *0.05; Figure 5). AUC was adjusted by regression model of the relationship between lnc-TCL6 and the covariates (age, sex) and demonstrated that lnc-TCL6 had poor discrimination performance when comparing the whole group of LC patients to healthy controls and HBV-infection groups; this was similar to the performance in discriminating CHB patients from healthy controls, HBV carriers from healthy controls, and CHB patients from HBV carriers (all *P *>* *0.05; Table 4).

**Table 3.** The characteristics of research participants in the validation phase

Variable	Healthy (*n* = 63)	HBV carrier (*n* = 32)	CHB (*n* = 28)	LC (*n* = 114)				*P*-value¹	*P*-value²
				All (*n* = 114)	CP-A (*n* = 49)	CP-B (*n* = 34)	CP-C (*n* = 31)		
Age, years	34.8 ± 14.8	37.5 ± 8.7	38.4 ± 11.4	50.1 ± 11.7	49.9 ± 10.4	52.2 ± 11.9	48.3 ± 13.3	<0.001	<0.001
Female gender	35 (55.6)	9 (28.1)	9 (32.1)	28 (24.6)	11 (22.4)	10 (29.4)	7 (22.6)	<0.001	0.023
WBC, × 10^9^/L	6.4 ± 1.5	6.5 ± 1.6	6.3 ± 1.5	4.6 ± 2.2	4.9 ± 2.4	3.9 ± 1.8	4.7 ± 2.4	<0.001	<0.001
ALT, U/L	16.0 [11.5–24.0]	31.5 [23.8–46.0]	208.0 [74.0–560.8]	28.0 [21.0–37.8]	28.0 [21.3–37.0]	27.0 [21.0–33.3]	32.5 [18.8–98.0]	<0.001	<0.001
Serum albumin								<0.001	<0.001
>35 g/L	16 (100.0)	26 (100.0)	13 (56.5)	60 (52.6)	44 (89.8)	10 (29.4)	6 (19.4)		
28–35 g/L	0	0	9 (39.1)	47 (41.2)	5 (10.2)	24 (70.6)	18 (58.1)		
<28 g/L	0	0	1 (4.3)	7 (6.1)	0	0	7 (22.6)		
Total bilirubin								<0.001	<0.001
>51 µmol/L	0	3 (10.3)	20 (90.9)	32 (28.1)	0	5 (14.7)	27 (87.1)		
34–51 µmol/L	0	0	1 (4.5)	14 (12.3)	3 (6.1)	9 (26.5)	2 (6.5)		
<34 µmol/L	12 (100.0)	26 (89.7)	1 (4.5)	68 (59.6)	46 (93.9)	20 (58.8)	2 (6.5)		

Values presented as mean ± standard deviation, median [interquartile range], or *n* (%).

HBV, hepatitis B virus; CHB, chronic hepatitis B; LC, liver cirrhosis; CP, Child–Pugh classification; SD, standard deviation; WBC, white blood cell; ALT, alanine aminotransferase.

*P*-value^1^ = comparison among Healthy, HBV carrier, CHB, and LC groups.

*P*-value^2^ = comparison among Healthy, HBV carrier, CHB, LC CP-A, LC CP-B, and LC CP-C groups.

**Table 4.** The diagnostic value of lnc-TCL6 in the validation set

Comparison	AUC	95% CI	*P*-value	Sensitivity	Specificity
Healthy vs HBV carriers	0.513	0.406–0.620	0.826	0.037	0.683
HBV carrier vs CHB	0.517	0.368–0.663	0.848	0.762	0.407
Healthy vs CHB	0.504	0.393–0.615	0.948	0.048	0.714
Healthy vs LC	0.512	0.436–0.587	0.801	0.772	0.318
HBV carrier vs LC	0.520	0.434–0.605	0.698	0.202	1.000
CHB vs LC	0.520	0.432–0.607	0.730	0.807	0.000

HBV, hepatitis B virus; CHB, chronic hepatitis B; LC, liver cirrhosis AUC, area under the curve; CI, confidence interval.

### Predictive ability of lnc-TCL6 in different clinical stages of HBV-related LC

**Figure 6.**
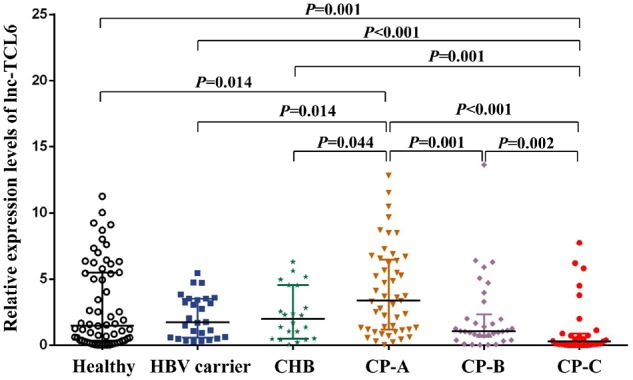
Differential expression levels of lnc-TCL6 in the three different clinical stages of liver cirrhosis (LC). Compared with the healthy controls, HBV carriers, and chronic hepatitis B (CHB) patients, the expression of lnc-TCL6 in Child–Pugh A (CP-A) cirrhosis was significantly elevated but gradually decreased through CP-B to CP-C cirrhosis. There was a minimum expression of lnc-TCL6 in the CP-C cirrhosis group compared with the other five groups. Data are presented as median with interquartile range.

Considering the diversity of clinical features of patients with cirrhosis, we divided the entire LC group into three classes according to CP score for further research and verified the corresponding diagnostic and predictive values of lnc-TCL6. The clinical characteristics such as age and sex were also significantly different between the between LC subgroups (CP-A, CP-B, CP-C) and healthy controls (Table 3). The expression level of lnc-TCL6 in patients with CP-A cirrhosis was significantly elevated compared with the healthy controls (*P *=* *0.014), HBV carriers (*P *=* *0.014), and CHB patients (*P *=* *0.044) but gradually declined through CP-B to CP-C (Figure 6). In addition, in CP-C patients, the relative lnc-TCL6-expression level (0.28 [0.04, 0.88]) was remarkably reduced compared with the other five groups (all *P *<* *0.01; Figure 6).

**Figure 7.**
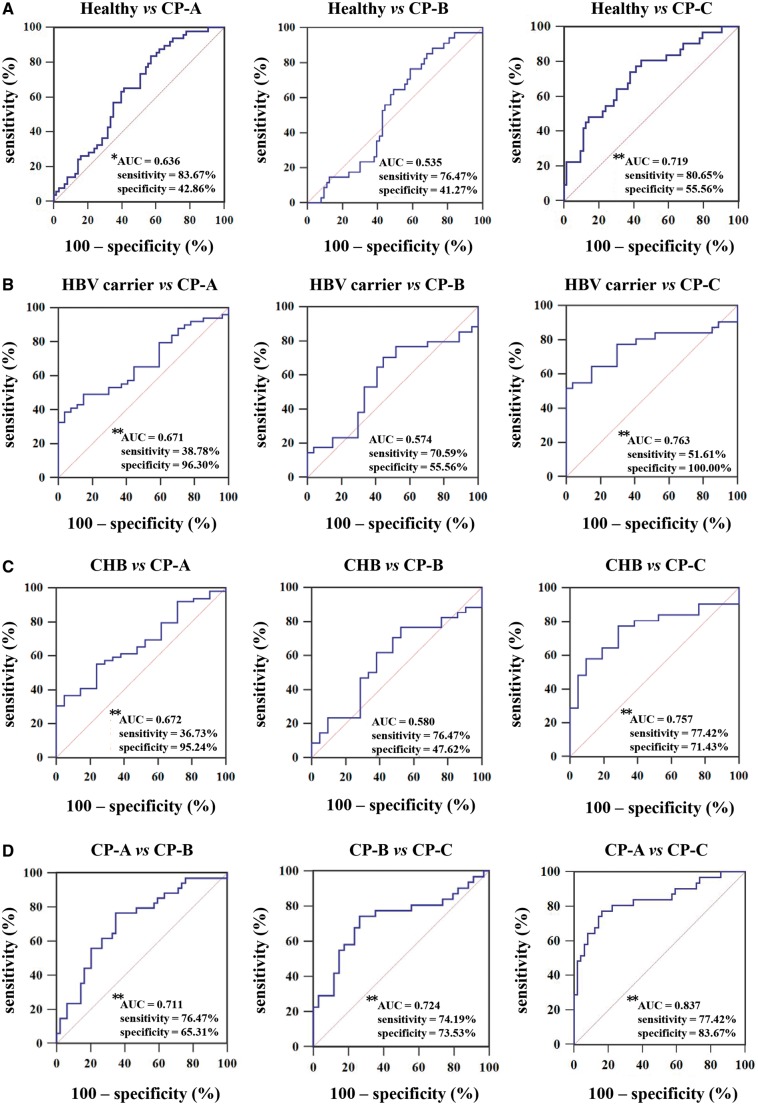
Receiver-operating characteristic (ROC) curve analysis of the diagnostic performance of lnc-TCL6 in the validation set. ROC plots for lnc-TCL6 discriminating Child–Pugh A (CP-A), CP-B, and CP-C cirrhosis from the healthy controls (A), HBV carriers (B), and chronic hepatitis B (CHB) patients (C), respectively, and among CP-A, CP-B, and CP-C cirrhosis (D). *P <* *0.05; **P <* *0.01.

Subsequently, the results demonstrated by covariate-adjusted ROC curves showed that lnc-TCL6 had high accuracy in discriminating patients with CP-A and CP-C from the healthy group (AUC = 0.636 and 0.719, respectively, both *P *<* *0.01; Figure 7A). Similarly, compared to the HBV-infection group, lnc-TCL6 also showed good diagnostic power for CP-A and CP-C patients (AUC for CP-A from HBV carriers and CHB were 0.671 and 0.672, respectively; AUC for CP-C from HBV carriers and CHB were 0.763 and 0.757, respectively; all *P *<* *0.01; Figure 7B and C).

Additionally, ROC curves were employed to observe the diagnostic capacity of lnc-TCL6 in distinguishing among the three CP classes of cirrhosis. The AUC was 0.711 for CP-B compared to CP-A, 0.724 for CP-C compared to CP-B, and 0.837 for CP-C compared to CP-A (all *P *<* *0.01, Figure 7D). The detailed 95% confidence intervals (CIs) and *P*-values of lnc-TCL6 in discriminating different cirrhosis CP classes is demonstrated in Supplementary Table 3. The results indicated that the diagnostic performance of lnc-TCL6 was independent of disease status, which made it an optimal diagnostic tool to identify different clinical stages of cirrhosis.

## Discussion

Histological LC is frequently indolent, asymptomatic, and undetected until complications of liver disease present. Current methods for the diagnosis of LC fall into two main categories: imaging and liver biopsy. The diagnostic abilities and predictive power of CT, MRI, ultrasonography, and liver biopsy are limited for unobvious clinical presentations [20]. Elasticity measurement (Fibroscan) is a promising technique for diagnosing early LC, but the examination is limited by ascites, morbid obesity, and small intercostal spaces [21]. By the way, serum hyaluronic acid, type III procollagen (PCIII), N-terminal propeptide of type III procollagen (PIIINP), type IV collagen (C-IV), and laminin (LN) levels have been used for many years as serum markers for LC diagnosis and screening. Although they can in part reflect the degree of fibrosis, the stage classification of fibrosis is too simple and the accuracy is influenced by liver inflammation [22, 23]. Aspartate transaminase-to-platelet ratio index, as a non-invasive tool to detect LC, have been extensively reviewed [24]. However, the results shown in other articles did not seem to meet the consistent criteria for an ideal surrogate fibrosis marker in LC [25–27].

Recently, the discovery of abnormal expression of lncRNAs in LC tissues paved the way for the analysis of lncRNAs for the diagnosis of cirrhosis. In our current study, we revealed that lnc-TCL6 (located on chromosome 14, NC_000014.9) in whole blood was a potential lncRNA marker for differentiating patients with HBV-related LC from those with CHB. The expression profile of lnc-TCL6 has been reported in the previous study as a candidate gene potentially involved in leukemogenesis [28]. Its potential role in the pathway of clear cell renal cell carcinoma (ccRCC) has also been described by a few studies [29, 30]. Interestingly, in our study, we found that the significant overexpression of lnc-TCL6 in the early clinical stage of LC (CP-A) showed a high predictive power for the occurrence of cirrhosis and the expression of lnc-TCL6 gradually declined with LC progress and was especially markedly down-regulated in the final stage of LC (CP-C). The results made lnc-TCL6 a potential diagnostic tool that could not only distinguish patients with early-stage cirrhosis from healthy people, but also discriminate among different clinical stages in cirrhosis patients.

Numerous lncRNAs are released to peripheral blood when liver-cell injury occurs and the expression levels of lncRNAs vary due to differences in liver inflammation and fibrosis [31]. Although the expression level of lnc-TCL6 in the whole HBV-related LC group was not significantly different from that in the healthy controls and HBV-infection group in our present research, the expression profile of lncRNAs should be explored in cirrhosis at various stages. The Child–Pugh classification can both characterize the degree of liver injury and predict the development of complications [20]. The total range of points (5–15) is not equally distributed across CP-A, CP-B, and CP-C cirrhosis, and it more efficiently mirrors the clinical impact of each grade regarding prognosis [32]. The diagnostic performance of lnc-TCL6 in different clinical stages of cirrhosis was first evaluated in our study. As widely used tools for the assessment of prognosis in LC, the model for end-stage liver disease (MELD) score and Child–Pugh score were heterogeneous under some specific conditions. The expression status of lnc-TCL6 in different stages of cirrhosis according to MELD score needs in-depth research.

To our knowledge, we are the first to report that the lnc-TCL6-expression profile was present not only in the overall group, but also in the different clinical stages of HBV-related LC in a large cohort. Of note, lnc-TCL6 could detect early-stage LC (CP-A) with a sensitivity of 83.67% compared with healthy controls and with a specificity of >95% compared with HBV-infection patients (including HBV carrier and CHB) through the analysis of ROC curves. Lnc-TCL6 showed 74.19%–77.42% diagnostic sensitivity and 65.31%–83.67% specificity for detecting three clinical stages of LC (CP-A, CP-B, and CP-C). However, the diagnostic power was significantly weaker in discriminating HBV-infection patients from healthy controls. This suggested that differential expression of lnc-TCL6 is related to various stages of LC but not HBV infection, and the functions of lnc-TCL6 in the process of LC should be investigated further. As the number of participants was relatively limited, replication of our findings in a multi-centre, large, prospectively studied cohort is required to validate our results in a further study.

In conclusion, the data indicated that lnc-TCL6 is a sensitive biomarker for early diagnosis of LC and a potential predictor of LC progression.

## Authors’ contribution

Study concept and design: L.J.L. and B.W. Performed the experiments: L.J.L., X.Y.W., S.W.T., H.L.L., and J.J. Recruited patients and collected specimens: X.M.P., S.W.P., and H.J.L. Data collection: L.J.L. and W.R.N.B. Statistical analysis: L.J.L., X.Y.W., Z.J.X., and B.W. Drafted the manuscript: L.J.L. and B.W. All authors read and approved the final manuscript.

## Funding

The study was supported in part by grants from the National Natural Science Foundation of China [U1501224], the Natural Science Foundation Team Project of Guangdong Province [2018B03031200], the Science and Technology Developmental Foundation of Guangdong Province [2017B020226003], and the Science and Technology Program of Guangzhou City [201604020118].

## Supplementary Material

goz050_Supplementary_Data
