# Supplementary material for: Lnc-TCL6 is a potential biomarker for early diagnosis and grade in liver-cirrhosis patients
Source: Gastroenterol Rep (Oxf). 2019 Oct 11;7(6):434–43. doi: 10.1093/gastro/goz050 (PMC6911997; doi:10.1093/gastro/goz050)
Supplement: goz050_Supplementary_Data [file goz050_supplementary_data.docx]

**Supplementary Table 1**. Primer sequences used in qRT-PCR

| **Genes** | **Forward primer** **(5’-3’)** | **Reverse primer** **(3’-5’)** |
| --- | --- | --- |
| ENST00000589723.1 | AAGGTTTAGAGTGAGGCTTCCA | TGACCAGGCTATTTCCACAGAT |
| Lnc-CTA-250D10.23 | CCCAGAGCCGTCACAT | CGGGTCATTTCTTCCCT |
| ENST00000602692.1 | GAAAGGGACTTGTGAGGTG | TGGGTGAAGATGTTAGGGA |
| ENST00000527317.2 | TGGGAGTCTTACTGAAATAGC | GCACATGGACCTGAAACAT |
| TCONS_00023502 | AGACCAGGGAACTGAAACA | AGGAGGAGGTGAGTGAGAC |
| Lnc-TCL6 | GCTGTCTAAGGGCTCATC | GGAGAAAGGCAAAGAACA |
| β-actin | CATGTACGTTGCTATCCAGGC | CTCCTTAATGTCACGCACGAT |

**Supplementary Table 2**. Candidate lncRNAs selection by microarray results.

| LncRNAs | AUC | 95% CI | *P*-value |
| --- | --- | --- | --- |
| ENST00000602692.1 | 0.141 | 0.000–0.352 | 0.016 |
| ENST00000589723.1 | 0.438 | 0.230–0.645 | 0.546 |
| Lnc-CTA-250D10.23 | 0.525 | 0.317–0.733 | 0.813 |
| ENST00000527317.2 | 0.438 | 0.145–0.730 | 0.674 |
| TCONS_00023502 | 0.438 | 0.143–0.732 | 0.674 |
| Lnc-TCL6 | 0.762 | 0.579–0.895 | 0.002 |

**Supplementary Table 3**. The characteristics of cirrhosis patients in the validation phase.

| Variable | Category | Value (*n* = 114) |
| --- | --- | --- |
| Age, years | - | 50.1 ± 11.7 |
| Sex, *n* (%) | Male | 86 (75.4) |
|  | Female | 28 (24.6) |
| White blood cell, ×10^9^/L | - | 4.6 ± 2.2 |
| Prothrombin time (prolong) | > 6 seconds | 28 (24.6) |
|  | 4–6 seconds | 18 (15.8) |
|  | < 4 seconds | 68 (59.6) |
| Ascites, *n* (%) | None | 76 (66.7) |
|  | Mild | 20 (17.5) |
|  | Moderate/severe | 18 (15.8) |
| Hepatic encephalopathy, *n* (%) | None | 108 (94.7) |
|  | 1–2 phase | 4 (3.5) |
|  | 3–4 phase | 2 (1.8) |
| Albumin, *n* (%) | > 35 g/L | 60 (52.6) |
|  | 28–35 g/L | 47 (41.2) |
|  | < 28 g/L | 7 (6.1) |
| Total bilirubin | > 51 μmol/L | 32 (28.1) |
|  | 34–51 μmol/L | 14 (12.3) |
|  | < 34 μmol/L | 68 (59.6) |
| Child-Pugh classification, *n* (%) | Class A | 49 (43.0) |
|  | Class B | 34 (29.8) |
|  | Class C | 31 (27.2) |

| Comparison | AUC | 95% CI | *P-*value |
| --- | --- | --- | --- |
| Healthy *vs* CP-A | 0.636 | 0.539–0.724 | 0.009 |
| Healthy *vs* CP-B | 0.535 | 0.431–0.637 | 0.554 |
| Healthy *vs* CP-C | 0.719 | 0.617–0.807 | <0.001 |
| HBV carrier *vs* CP-A | 0.671 | 0.554–0.775 | 0.005 |
| HBV carrier *vs* CP-B | 0.574 | 0.441–0.700 | 0.329 |
| HBV carrier *vs* CP-C | 0.763 | 0.634–0.865 | <0.001 |
| CHB *vs* CP-A | 0.672 | 0.549–0.779 | 0.009 |
| CHB *vs* CP-B | 0.580 | 0.439–0.712 | 0.322 |
| CHB *vs* CP-C | 0.757 | 0.618–0.865 | <0.001 |
| CP-A *vs* CP-B | 0.711 | 0.601–0.805 | <0.001 |
| CP-B *vs* CP-C | 0.724 | 0.599–0.828 | <0.001 |
| CP-A *vs* CP-C | 0.837 | 0.737–0.910 | <0.001 |

**Supplementary Table 4**. The diagnostic value of lnc-TCL6 in discriminating different cirrhosis groups.

HBV, hepatitis B virus; CHB, chronic hepatitis B; CP, Child-Pugh classification
